# Cholera Toxin Enhances Vaccine-Induced Protection against Mycobacterium Tuberculosis Challenge in Mice

**DOI:** 10.1371/journal.pone.0078312

**Published:** 2013-10-23

**Authors:** Kristin L. Griffiths, Elena Stylianou, Hazel C. Poyntz, Gareth J. Betts, Helen A. Fletcher, Helen McShane

**Affiliations:** Jenner Institute, University of Oxford, Oxford, United Kingdom; University of Cape Town, South Africa

## Abstract

Interleukin (IL)-17 is emerging as an important cytokine in vaccine-induced protection against tuberculosis disease in animal models. Here we show that compared to parenteral delivery, BCG delivered mucosally enhances cytokine production, including interferon gamma and IL-17, in the lungs. Furthermore, we find that cholera toxin, delivered mucosally along with BCG, further enhances IL-17 production by CD4^+^ T cells over mucosal BCG alone both in the lungs and systemically. This boosting effect of CT is also observed using a vaccine regimen of BCG followed by the candidate vaccine MVA85A. Using a murine *Mycobacterium tuberculosis* (*M.tb*) aerosol challenge model, we demonstrate the ability of cholera toxin delivered at the time of a priming BCG vaccination to improve protection against tuberculosis disease in a manner at least partially dependent on the observed increase in IL-17. This observed increase in IL-17 in the lungs has no adverse effect on lung pathology following *M.tb* challenge, indicating that IL-17 can safely be boosted in murine lungs in a vaccine/*M.tb* challenge setting.

## Introduction

Tuberculosis (TB) remains a major global health threat, with an annual worldwide incidence of approximately nine million [1]. The only licensed vaccine, Bacille Calmette Guérin (BCG), is currently administered at birth throughout most of the world, however its efficacy, particularly in TB endemic countries, is highly variable [2]. There is therefore an urgent need for a more effective TB vaccine regimen. Protection against TB disease is known to depend at least partially on interferon (IFN)-γ, although it is clear that a strong IFN-γ-based T cell response is not sufficient to confer complete protection. Interleukin (IL)-17 is emerging as an important cytokine in vaccine-induced protection against TB disease in both mice [3], [4] and cattle [5]. Work in murine TB models has found that IL-17 expression in the lungs mediates the formation of lymphocytic granulomas through inducing the upregulation of the chemokine ligand CXCL13 and thus the recruitment of highly activated T cells bearing the CXCL13 receptor, CXCR5 [6]. Although its importance in humans is not yet known, IL-17 is readily detectable as a product of T cells isolated from both bronchoalveolar lavage fluid and pleural fluid from patients with active TB [7]–[10].

MVA85A is a modified Vaccinia virus Ankara expressing the mycobacterial antigen 85A (Ag85A). The MVA85A vaccine is used to boost CD4^+^ T cell responses primed by BCG vaccination [11]. The vaccine has been administered to over 3000 humans over the course of the past decade, with good safety and high levels of antigen-specific T cells induced in adults. The vaccinated groups include adults in the UK and in the Gambia, South Africa and Senegal, as well as individuals with latent TB infection, adolescents, infants and HIV-infected adults. A Phase IIb efficacy trial of MVA85A in South African infants has recently been completed [12], and a second Phase IIb trial in HIV-infected adults in South Africa and Senegal is currently underway (ClinicalTrials.gov identifier: NCT01151189). Results from the efficacy trial in infants showed no difference in TB incidence between infants vaccinated with BCG alone or BCG followed by MVA85A [13], however given that the IFN- γ responses in infants were lower than those observed in adults, continued work on MVA85A is warranted with potential in populations where immunity is higher. The primary readout for immunogenicity is IFN-γ production measured by ELISpot, which has been shown to be produced predominantly by CD4^+^ T cells [11]. Analysis of cytokine expression in both whole blood and PBMC from vaccinees by flow cytometry has also shown induction of IL-2, TNF-α and IL-17 in CD4^+^ T cells by MVA85A [14]–[16].

Cholera toxin (CT), produced by the bacterium *Vibrio cholerae*, is a potent IL-17-inducing adjuvant, which acts through the cyclic adenosine monophosphate (cAMP) pathway. It exists as a holotoxin, with a pentameric B subunit that binds to the ganglioside GM1 on the cell surface. This binding facilitates translocation of the A subunit through the endoplasmic reticulum and into the cytosol where it binds ADP ribosylation factor 6, which ribosylates adenylyl cyclase (AC), rendering it constitutively active, resulting in increased levels of cAMP in the cytosol and therefore an increase in events downstream of cAMP [17]. With respect to modulation of the immune function of cells, CT has been shown to have a direct negative effect on IL-12 production by preventing binding of IRF (interferon regulatory factor) 8 to the IL-12p40 promoter in dendritic cells (DCs) [18]. Furthermore, increased intracellular cAMP in DCs, induced either through CT treatment or through administration of the cell-permeable dibutyryl cAMP or the cAMP-inducing agent forskolin, increased IL-17 production by CD4^+^ T cells following culture with the treated DCs and was found to act through the protein kinase A (PKA) pathway [19], inducing IL-17 in an IL-6 and IL-1β-dependent manner. CT has previously been used as an adjuvant in murine models and been shown to be a potent inducer of IL-17, conferring protection against IL-17-dependent diseases [19], [20].

A CT-like toxin, *Escherichia coli*'s heat labile toxin, has been used in a human vaccine trial as an adjuvant for intranasally-delivered vaccines against *M.tb* and HIV. The trial had to be terminated, however, due to the development of a Bell's palsy in two of the volunteers [21]. CT cannot therefore be used as an adjuvant in human vaccines, however alternatives that target the cAMP pathway could be evaluated.

Here, we aim to investigate the potential for a BCG – MVA85A vaccine regime to induce IL-17 in a model murine system in order to then investigate how boosting IL-17 might affect *M.tb* challenge outcome. We show that BCG – MVA85A delivered intranasally does indeed induce IL-17 both mucosally and systemically and that CT can enhance IL-17 production when administered together with BCG. When challenged with *M.tb*, these mice are better protected than those not receiving CT, suggesting that the cAMP pathway induces responses important for protection against TB disease.

## Materials and Methods

### Animals and vaccinations

All animal experiments were carried out in accordance with the guidelines set out by the UK Home Office in the Animals (Scientific Procedures) Act 1986 and were approved under the Jenner Institute project license 30/2889. Experiments were carried out under the personal license 30/8774. Maximum care was taken to ensure minimal suffering.

Six to eight week old Balb/c mice were obtained from Harlan, UK.

BCG Pasteur (passaged in-house) was administered at 4×10^5^ CFU/vaccination. MVA85A was administered at 1×10^6^ PFU/vaccination. Cholera toxin (List Labs; Campbell, USA) was administered at 2 µg/mouse in 50 µL mixed with the vaccine.

The anti-IL-17A blocking antibody (MAB421) and its corresponding IgG2a isotype control (MAB006), both from R&D Systems (Minneapolis, USA), were administered intraperitoneally (*i.p.*) at 100 µg/mouse in 100 µL every three days following challenge for four weeks.

For intranasal (*i.n.*) vaccination, animals were sedated using IsoFlo (Oxford University Veterinary Services; UK) and the vaccine administered in a 50 µL bolus using a pipette held over the nose.

Intradermal (*i.d.*) vaccinations of 25 µL into each ear dorsum using a U-100 29G needle were administered under IsoFlo anaesthesia.

Intraperitoneal administration of substances was done on restrained animals and delivered in 100 µL using a 1 mL syringe with a 29G needle.

### *M.tb* aerosol challenge

Animals were challenged using a Biaera AeroMP-controlled nebuliser contained in a Category Level 3 TCOL isolator. Erdmann *M.tb* (BEI Resources; Manassas, USA)) was made up to 5×10^6^ CFU/mL in PBS and transferred to the nebuliser in the isolator. Animals were loaded into nose-only restrainers and fitted to the exposure unit. The programme was run for 10 min plus a 5 min purge with the airflow set to 12 L/min at a pressure of 20psig. The target dose of 100–200 CFU/animal was confirmed by sacrificing two unvaccinated animals from each run 24 hours post-challenge.

### Organ harvest and stimulation for immunogenicity

For immunogenicity studies, spleens and lungs were aseptically removed. Prior to dissection, lungs were perfused using 5 mL PBS passed through the right ventricle. Spleens were passed through a 70 µm sieve into 5 mL Dulbecco's modified Eagle Medium (Sigma Aldrich; St Louis, USA) supplemented with 2 mM L-glutamine and 100 U Penicillin/100 µg Streptomycin (both from Sigma Aldrich) and transferred to a 15 mL tube. Splenocytes were treated with red blood cell lysis buffer.

Lungs were minced and digested using a DNase/collagenase mix containing 1.4 mg/mL collagenase (Sigma Aldrich) and 60 µg DNase (Sigma Aldrich), followed by red blood cell lysis.

Cells were plated in a round bottom 96 well plate and an Ag85A peptide pool or PPD added to the relevant wells to give a final concentration of 2 µg/mL for the peptides and 10 µg/mL for the PPD. Brefeldin A (Sigma Aldrich; 25 µg/well) and 0.7 µL/well GolgiStop (BD Pharmingen; Franklin Lakes, USA) were added two hours after the addition of the antigen and cells were stimulated for a further 16 hours.

Following stimulation, cells were stained with Live/Dead Fixable Dead Cell Stain (Invitrogen; Grand Island, USA) followed by a surface staining cocktail, including antibodies (all from eBioscience (San Diego, USA) except where indicated) against CD4-Qdot605 (Invitrogen; GK1.5), B220-PE-Texas Red (RA3-6B2) and CD19-Pacific Blue (eBio1D3). Following permeabilisation using CytoFix/CytoPerm (BD Biosciences), cells were stained intracellularly for analysis of cytokine production. Antibodies for intracellular staining included CD3-PE-Cy5 (145-2C11), CD8-APC-AlexaFluor780 (53–6.7), IFN-γ-AlexaFluor488 (XMG1.2) and IL-17A-PE (eBio17B7). Samples were analysed using an LSR II flow cytometer and data processed using FlowJo (TreeStar; Ashland, USA).

### Organ harvest for bacterial load quantitation

Lungs and spleens removed from challenged animals were placed in reinforced 2 mL tubes containing 2.8 mm ceramic beads (Stretton Scientific; Stretton, UK)). Organs were homogenised at 5500 rpm for 20 seconds using a Precellys 24 (Stretton Scientific) tissue homogeniser before diluting and plating on Middlebrook 7H10 agar plates. Plates were counted four weeks after plating.

Plates were made using Middlebrook 7H10 Agar Base (Sigma Aldrich) with 2.5 mL glycerol added/500 mL. After autoclaving for sterilisation, 50 mL OADC (BD Diagnostic Systems) enrichment was added/500 mL and plates poured at 15–20 mL agar/plate.

### Organ preparation for histology

For histology, animals were sacrificed by cervical dislocation. Spleen and the other lung lobe were removed and placed in tubes as described above for CFU quantitation. A hole was cut in the trachea using a blade and 500 µL 10% Neutral Buffered Formalin (NBF; Sigma Aldrich) injected using 0.3 mm×0.6 mm tubing attached to a 23G needle on a 2 mL syringe. The lung was removed and placed in 7.5 mL 10% NBF overnight. The following day the lobes were transferred to 70% ethanol and stored at 4°C. Embedding, sectioning, and haematoxylin and eosin (H&E) staining were performed by Histopathox (Oxford, UK).

### Analysis of lung sections

Stained sections were scanned by the Oxford Centre for Histopathology Research (OCHRe; Oxford, UK) at ×20 magnification using a Hamamatsu slide scanner. Files were provided as .ndpi files, which were converted to .tif files using the ndip2tiff software provided by Dr Christophe Deroulers (NDPITools; http://www.imnc.in2p3.fr/pagesperso/deroulers/software/ndpitools/) [22]. The k means clustering algorithm in MATLAB (MATLAB 7.13.0, The MathWorks Inc., Natick, USA http://www.mathworks.com.au/help/stats/kmeans.html) was used to determine exclusive k means clustering of three colours (white, pink and purple) in the haematoxylin and eosin-stained tissue. Each cluster (colour) was further defined by selecting 10 hues for each. This produced a representative image composed of only the three colours selected, with each pixel being assigned to one of the three colours. Finally, a statistical module was programmed in order to determine the percentage contribution of each cluster to the overall image by counting the number of pixels belonging to each colour group.

### Statistical analyses

Analysis of two data sets was performed using a t test (parametric) or Mann-Whitney test (non-parametric). One way ANOVA was used for comparing three or more groups of normally distributed data followed by post-hoc tests. Analyses were performed using statistical analysis tools in GraphPad Prism version 5.0 for Mac OSX.

## Results

### Intranasal delivery of BCG + CT enhances IL-17 and improves vaccine efficacy following *M.tb* challenge

**Figure 1:**
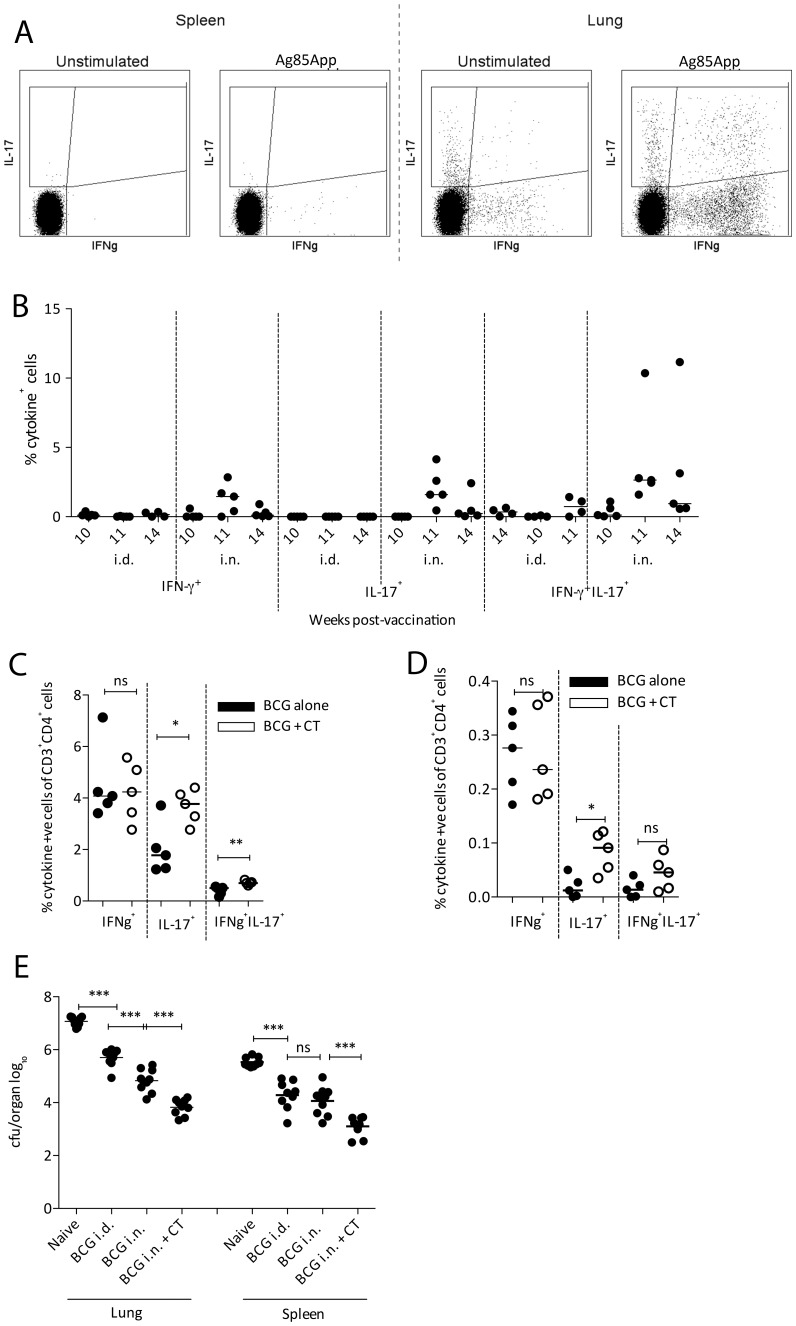
Effect of cholera toxin on BCG immunogenicity and *M.tb* challenge outcome. (**A**) Cytokine gating in spleens and lungs, unstimulated vs. Ag85A peptide pool-stimulated. (**B**) Balb/c mice received 4×10^5^ CFU BCG *i.n.* or *i.d.* followed 10 weeks later by 1×10^6^ PFU MVA85A *i.d.* At weeks 10, 11 and 14 post-BCG, lungs were examined for cytokine production by ICS following Ag85A peptide pool stimulation in the presence of Brefeldin A and GolgiStop. (**C** & **D**) Balb/c mice received 4×10^5^ CFU BCG±2 µg CT *i.n*. After 10 weeks, lungs and spleen were dissected and stimulated with PPD in the presence of Brefeldin A and GolgiStop. Percentages of CD4^+^ T cells producing IFN-γ and IL-17 were calculated following ICS on lung cells (**C**) and splenocytes (**D**). Mice receiving BCG only are plotted with closed circles and those receiving CT have open circles. P values were calculated using a Mann Whitney test (n = 5). (**E**) Ten weeks post-BCG, mice received ∼100 CFU *M.tb* via aerosol and four weeks later lungs and spleen were homogenised and plated out for CFU quantitation. Statistical analysis was performed using a one way ANOVA and post-hoc tests on the vaccinated groups (n = 8).

As previously reported, in mouse models of TB disease, intranasal (*i.n.*) delivery of BCG improves protection against *M.tb* challenge compared to intradermal (*i.d*.) delivery [23]–[26]. As shown in Figure 1B, using a vaccine regimen of BCG followed by MVA85A (BCG – MVA85A) we have found that *i.n.* vaccination enhances overall cytokine production in the lungs compared to *i.d.* vaccination. Furthermore, production of IL-17 by CD4^+^ T cells in lungs is enhanced following *i.n.* BCG and MVA85A boost (Fig 1B). We have therefore based the experiments shown in this paper on *i.n.* delivery of the vaccine. Sample flow plots for lungs and spleen are shown in Figure 1A.

In order to investigate the effect of CT on BCG immunogenicity, Balb/c mice were vaccinated with BCG *i.n.* Half the animals received CT (2 µg/mouse) co-administered with the BCG. Lungs and spleen were taken 10 weeks post-vaccination and responses to purified protein derivative (PPD) determined by intracellular cytokine staining (ICS). Balb/c mice were chosen due to the existence of an I-A^d^-restricted epitope (p15) in Ag85A for later experiments involving MVA85A; furthermore, their relative resistance to *M.tb* infection allows establishment of a long-term infection.

Production of IFN-γ by CD4^+^ T cells in either lungs (Fig 1C) or spleen (Fig 1D) was not affected by CT treatment. Percentages of cells producing IL-17 in both lungs and spleen, however, were significantly increased in the group receiving BCG + CT (Fig 1C & D; p = 0.0317 and 0.0159 for lung and spleen respectively). Percentages of CD4^+^ T cells simultaneously producing IFN-γ and IL-17 in the lungs, but not spleen, were significantly increased following CT treatment (p = 0.0079).

These vaccination regimens were further investigated in an *M.tb* aerosol challenge model in order to evaluate the effect of CT on vaccine-induced protection following *M.tb* exposure. Balb/c mice vaccinated with BCG±CT *i.n.* were challenged with *M.tb* via aerosol 10 weeks post-BCG. Lung and spleen bacterial burdens (measured in colony forming units (CFU)) were determined four weeks post-challenge. As shown in Figure 1E, results following *M.tb* challenge confirmed BCG-conferred protection (mean (log_10_)  = 7.07 and 5.71 CFU/lung for naïve and BCG *i.d.* respectively, p<0.0001). Delivery of BCG *i.n.* improved protection in the lungs compared to BCG *i.d.* (mean (log_10_)  = 5.71 and 4.83 CFU/lung for BCG *i.d.* and *i.n.* respectively, p = 0.0001). Similar results were observed in the spleen. Administration of CT together with BCG *i.n.* improved protection in the lungs further (mean (log_10_) = 4.83 and 3.83 CFU/lung for BCG *i.n.* and BCG + CT *i.n.* respectively, p<0.0001).

### Intranasal delivery of BCG + CT – MVA85A enhances IL-17 and improves efficacy following *M.tb* challenge

In order to investigate the ability of the novel TB vaccine MVA85A to further boost the enhanced IL-17 responses observed following BCG + CT administration *i.n*., Balb/c mice were vaccinated with BCG±CT *i.n.* followed 10 weeks later by MVA85A *i.n.*

**Figure 2:**
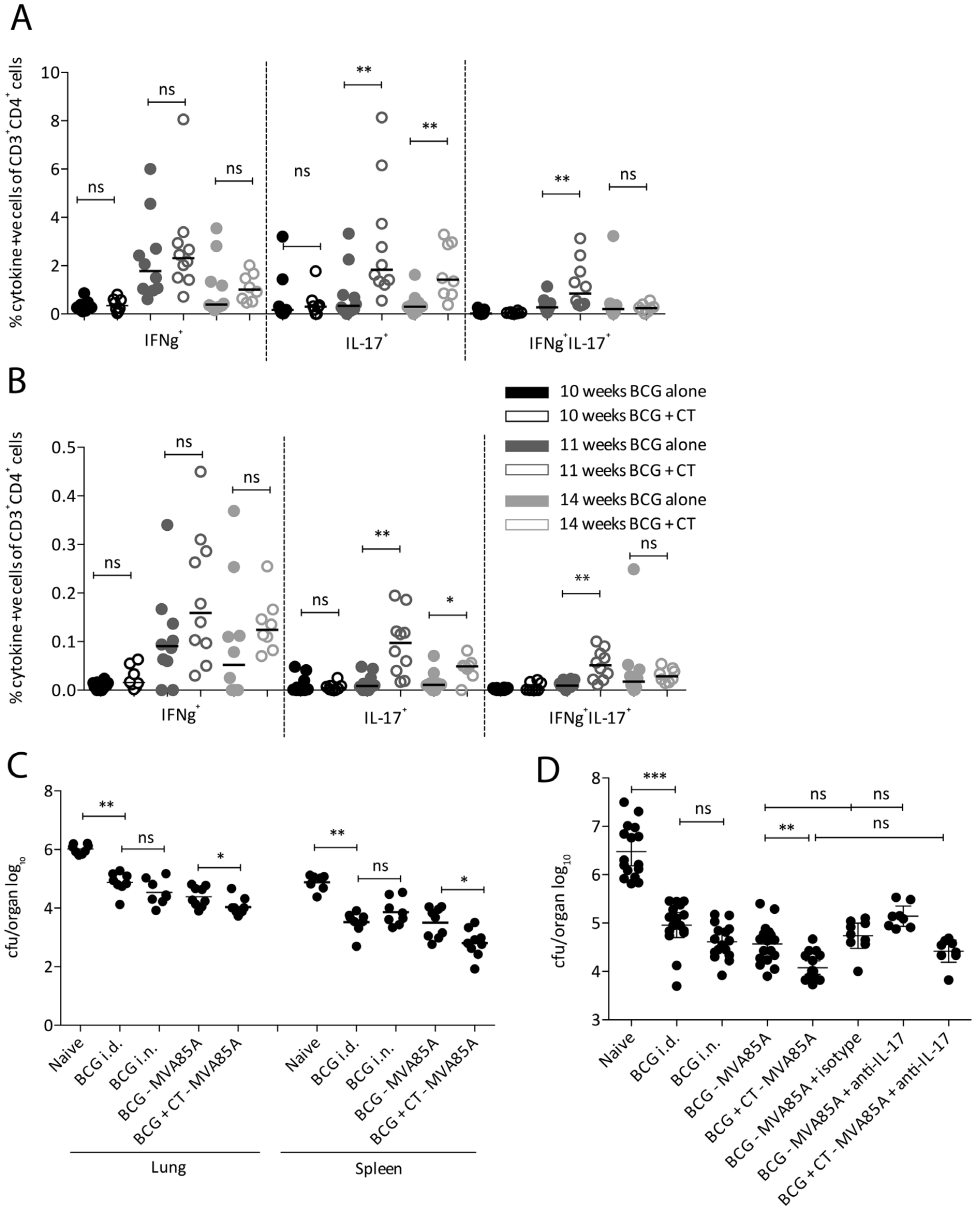
Immunogenicity and protective efficacy of BCG + CT – MVA85A. Balb/c mice received BCG±CT *i.n.* followed by 1×10^6^ CFU MVA85A 10 weeks later. Lungs (**A**) and spleen (**B**) were taken at 10 (black circles), 11 (dark grey circles) and 14 (light grey circles) weeks post-BCG and cytokine-producing cells responding to an Ag85A peptide pool quantified using ICS. Responses from animals receiving BCG – MVA85A (closed circles) were compared with those receiving BCG + CT followed by MVA85A (open circles). Statistical analysis was performed using a Mann Whitney test. n = 10, five each from two experiments. (**C**) Balb/c mice were vaccinated as above. Control groups included unvaccinated and BCG *i.d.* A group receiving BCG *i.n.* was included to compare BCG – MVA85A *i.n.* to BCG *i.n*. Animals were exposed to ∼100 CFU *M.tb* via aerosol four weeks post-MVA85A. Four weeks post-challenge, lungs and spleen were homogenised and plated for CFU quantitation. (**D**) Balb/c mice were vaccinated and challenged as described above. Groups receiving BCG – MVA85A and BCG + CT – MVA85A received an anti-IL-17 blocking antibody (MAB421; R&D Systems) administered *i.p.* every three days post-challenge. One group receiving BCG – MVA85A received an IgG2a isotype control antibody (MAB006; R&D Systems) on the same regimen. Mice were culled four weeks post-challenge and lung CFU quantitated as described above. Statistical analysis was performed using a one way ANOVA and post-hoc tests on the vaccinated groups (n = 8–16).

As observed for BCG + CT alone, boosting further with MVA85A (BCG + CT – MVA85A) did not significantly change percentages of IFN-γ-producing CD4^+^ T cells in the lungs (Fig 2A) or spleen (Fig 2B) compared to animals receiving only BCG – MVA85A, although there was a trend towards higher percentages of IFN-γ^+^ cells in animals receiving CT. Percentages of CD4^+^ T cells producing IL-17, in contrast, were significantly higher in animals vaccinated with BCG + CT – MVA85A compared to those receiving BCG – MVA85A (p = 0.0068 and 0.0021 at 11 and 14 weeks post-BCG respectively in lungs, and p = 0.0017 and 0.0362 at 11 and 14 weeks post-BCG respectively in spleen). For IFN-γ^+^IL-17^+^ cells, a significant increase following CT treatment was observed one week post-MVA85A (11 weeks post-BCG) in the lungs and the spleen (p = 0.0068 and 0.015 for lungs and spleen respectively). This was not detectable four weeks post-MVA85A.

Animals receiving the vaccine regimens described above were challenged with aerosolised *M.tb* four weeks post-MVA85A in order to investigate whether CT could enhance vaccine efficacy conferred by BCG – MVA85A. As shown in Figure 2C, BCG *i.d.* conferred the expected 1 – 2 log protection against TB disease in both lungs and spleen (mean (log_10_) in the lungs  = 6.02 and 4.87 CFU for naïve and BCG *i.d.* respectively). BCG delivered *i.n.* conferred slightly but not statistically significantly better protection in lungs compared to BCG *i.d.* This is in contrast to the results observed in Figure 1E, a factor that could be attributed to a variable effect dependent on factors such as time post-vaccination (10 weeks vs. 14 weeks) or *M.tb* challenge dose. The same was observed for animals vaccinated with BCG – MVA85A. When CT was administered along with BCG prior to MVA85A (BCG + CT – MVA85A), there were lower bacterial counts in both lungs and spleen compared to BCG – MVA85A, and these reached significance in the lungs and spleen (for lung: mean (log_10_)  = 4.39 and 4.04 CFU for BCG – MVA85A only and CT-treated respectively; for spleen: mean  = 3.50 and 2.81 CFU for BCG – MVA85A and CT-treated respectively).

We next attempted to block the improved protection conferred by CT co-administration using an anti-IL-17A blocking antibody, administered intraperitoneally every three days post-challenge for four weeks. The following groups received the blocking antibody: BCG – MVA85A and BCG + CT – MVA85A, all administered *i.n*. A group vaccinated with BCG – MVA85A received an IgG2A isotype control antibody.

As in the first experiment, BCG + CT – MVA85A significantly enhanced protection against aerosol challenge (Fig 2D; mean (log_10_)  = 4.77 and 4.12 CFU for BCG – MVA85A and CT-treated respectively). Administration of the isotype control antibody did not significantly affect the lung CFU indicating that any difference seen in the antibody-treated groups can be attributed to the antibody's specificity (mean (log_10_)  = 4.77 and 4.74 CFU for no antibody and isotype respectively). Comparing the BCG – MVA85A group receiving antibody and the group receiving the isotype control, the lung CFU was higher in the anti-IL-17-treated group, although this difference was not statistically significant using ANOVA followed by Bonferroni's multiple comparison post-test (mean (log_10_)  = 4.74 and 5.14 CFU for isotype and anti-IL-17 respectively). A similar trend was observed in animals receiving BCG + CT – MVA85A with the anti-IL-17 antibody compared to BCG + CT – MVA85A alone (mean (log_10_)  = 4.12 and 4.42 CFU for no antibody and anti-IL-17 treatment, respectively).

### Lung pathology is not adversely affected following CT treatment

**Figure 3:**
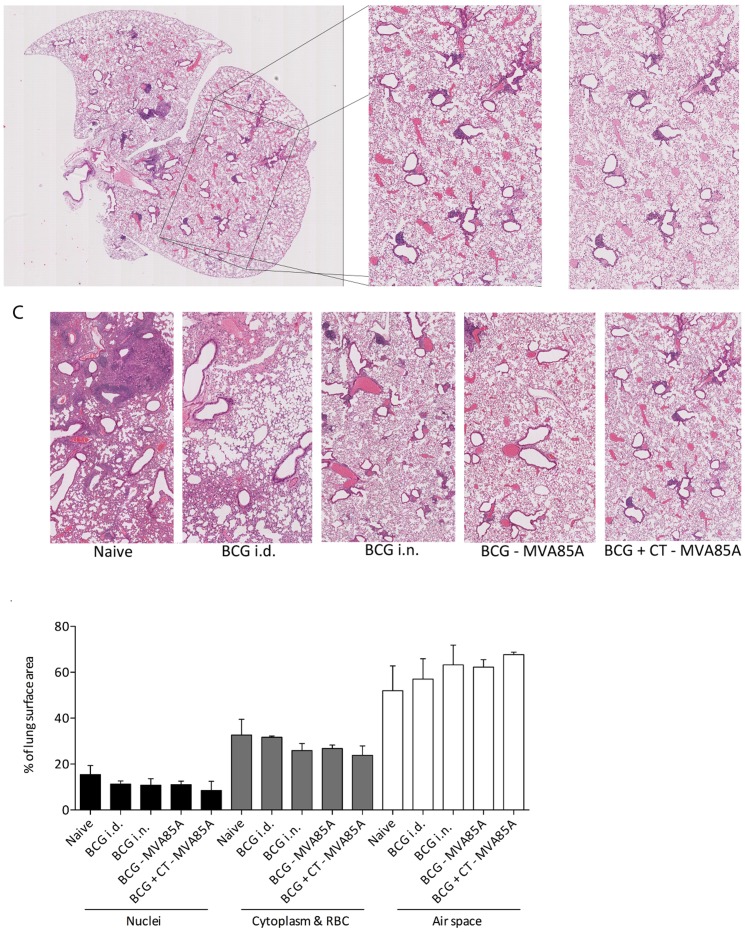
Histological analysis of *M.tb*-exposed lungs. Four Balb/c mice from each group shown in Figure 2C had one lung lobe inflated with 10% NBF and dissected. Lungs were embedded in paraffin, sectioned, mounted onto slides and stained with haematoxylin and eosin. Images were cropped so that no external white space was visible (**A**). Analysis was performed using a k means clustering algorithm in MATLAB (**B**). (**C**) Representative sections from each group. (**D**) Percentages of the image represented by one of three colours (purple, red or white) were calculated and plotted, n = 2-4.

Interleukin 17 is a highly inflammatory cytokine implicated as a negative factor in many inflammatory diseases such as multiple sclerosis, psoriasis and rheumatoid arthritis [27]-[29]. Aside from transient illness immediately after vaccination, no adverse effects were observed in animals receiving CT following *M.tb* challenge, however it was important to examine the lungs of the challenged animals in order to investigate the effects of the CT-induced IL-17 on lung pathology. One lung lobe was dissected from four animals in each group at the experimental endpoint (four weeks post-infection) and haematoxylin and eosin staining was used to examine the inflammatory state of the lung. Section images were cropped so that no white space was visible surrounding the section (∼30% of the image) as shown in Figure 3A.

Cropped sections were analysed using a k-means clustering algorithm in MATLAB in order to determine the percentage of the section represented by each of the following colours: purple (nuclei), pink (red blood cells and cytoplasm) and white (space for gas exchange). A sample of the resulting image in comparison to the original is shown in Figure 3B. Representative images from each group are shown in Figure 3C. Figure 3D shows the percentages of each ‘colour’ for each of the groups. Although numbers were too low to perform statistical analysis, there is a trend towards a decrease in cell infiltrate (as measured by % nuclei, and % red blood cells and cytoplasm) with a decrease in lung CFU, and an increase in airspace along with a decrease in lung CFU. Thus, the animals receiving CT had lower levels of cell infiltrate and more airspace compared with unvaccinated animals and animals receiving less effective vaccine regimens. None of these yielded a significant correlation when tested (data not shown).

## Discussion

Experiments described in this paper aimed to explore the use of CT as a proof-of-concept IL-17-inducing adjuvant in both BCG and BCG – MVA85A vaccine regimens with the aim of boosting IL-17 production by CD4^+^ T cells and investigating the resulting effects on vaccine efficacy in an *M.tb* aerosol challenge model. Study of vaccine-induced immunogenicity showed that CT administered *i.n*. along with BCG was able to induce high levels of IL-17 post-BCG in lungs and spleen. Furthermore, the immunogenicity of BCG – MVA85A was also boosted by co-administration of CT with the BCG prime. Following *M.tb* challenge, mice receiving BCG + CT (± MVA85A) had lower lung and spleen CFU compared to the animals receiving non-adjuvanted BCG. The effect in the lungs could be partially blocked by an anti-IL-17 blocking antibody administered post-challenge, supporting the already-published theory of a role for IL-17 in vaccine-mediated protection against TB disease [3], [4], [30]. Although not investigated here, other cytokines involved in the IL-17 pathway, such as IL-23, IL-21 and IL-22 are likely also upregulated by CT treatment and it is probable that these also play a role in protection following *M.tb* infection. Further work should define comprehensively the cytokine profile induced by CT. This work demonstrates a protective effect of IL-17 against short-term challenge. Further work is merited to evaluate the durability of this effect.

Given the delivery of BCG via the intranasal route, there could be some residual BCG presence in the lungs even 14-18 weeks following administration. This might confound the results both in terms of residual BCG contributing to the immune response to an *M.tb* challenge, and in terms of BCG being detected on the agar plates used to determine bacterial burden. *M.tb*-specific agar plates were not utilised in these experiments, however we would expect the levels of BCG present in the lungs to be equal across the groups, thus cancelling out this potential effect.

Adverse effects on lung pathology are a concern accompanying any study involving boosting of IL-17, due to its highly inflammatory profile. Work shown here suggests that establishment of a chronic infection in the lungs of animals vaccinated with a regimen shown to boost IL-17 has no adverse effects on lung pathology, however more work is needed to confirm this. In these experiments, although not statistically significant, lung pathology was improved with the use of a regimen that reduced increased levels of IL-17 pre-challenge and resulted in decreased bacterial load in the lungs.

Although CT is not an adjuvant suitable for use in humans due to its strong inflammatory nature and reported side effects, including possible neurological symptoms [21], its use in animal models is a valuable tool for studying a) the outcomes of such a potent IL-17-inducing adjuvant, and b) mechanisms that could be targeted to achieve a similar results. We have shown here that CT given at the time of a mucosal BCG prime is an adjuvant capable of improving vaccine-induced protection against murine *M.tb* challenge. Cholera toxin acts through the cAMP pathway, thus implicating this pathway as a potential target for adjuvants designed to be given with BCG either as a vaccine on its own or as a prime for a boosting vaccine such as MVA85A. Extracellular ATP is a potential molecule that could be explored for this purpose. Recent data has shown that mice lacking the ATP receptor P2X7 have worsened disease following *M.tb* challenge through a decrease in P2X7-mediated phago-lysosomal fusion [31]. Furthermore, P2X7 activation through ATP binding activates the NALP3 inflammasome, reviewed in [32], and ATP can also bind to the P2Y11 receptor [33], activating the cAMP pathway, both of which lead to induction of IL-17-inducing cytokines and could be exploited for induction of a protective immune response.

In conclusion, these data suggest that further evaluation of the mechanisms of improved protection in mice in parallel with evaluation of the mucosal route of vaccination and IL-17-inducing adjuvants is merited in humans.
